# Genome wide analysis of TLR1/2- and TLR4-activated SZ95 sebocytes reveals a complex immune-competence and identifies serum amyloid A as a marker for activated sebaceous glands

**DOI:** 10.1371/journal.pone.0198323

**Published:** 2018-06-21

**Authors:** Dániel Törőcsik, Dóra Kovács, Szilárd Póliska, Zita Szentkereszty-Kovács, Marianna Lovászi, Katalin Hegyi, Andrea Szegedi, Christos C. Zouboulis, Mona Ståhle

**Affiliations:** 1 Department of Dermatology, Faculty of Medicine, University of Debrecen, Debrecen, Hungary; 2 Unit of Dermatology and Venereology, Department of Medicine, Karolinska Institutet, Karolinska University Hospital, Stockholm, Sweden; 3 Department of Biochemistry and Molecular Biology, Genomic Medicine and Bioinformatics Core Facility, Faculty of Medicine, University of Debrecen, Debrecen, Hungary; 4 Division of Dermatological Allergology, Faculty of Medicine, University of Debrecen, Debrecen, Hungary; 5 Departments of Dermatology, Venereology, Allergology and Immunology, Dessau Medical Center, Brandenburg Medical School Theodore Fontane, Dessau, Germany; San Gallicano Dermatologic Institute, ITALY

## Abstract

Toll-like receptors (TLR) 2 and 4 are active in sebaceous glands and play a central role in the development of acne. Still, there is only limited knowledge on their effect on sebocytes. In this work we performed global gene expression profile analysis with functional clustering of the differentially regulated genes of TLR1/2 (PAM3CSK4)- and TLR4 (lipopolysaccharide [LPS])-activated SZ95 sebocytes. Both TLR1/2- and 4-activation promoted inflammation in a similar manner already at an early time-point (6 hours), regulating genes involved in inflammation, wound healing and chemotaxis reflecting a more complex cytokine and chemokine regulation than previously known. Importantly, lipid metabolism, the primary feature of sebocytes, was affected at the level of gene expression only at a later time point (24 hours) indicating that sebocytes prioritize to exert a pro-inflammatory phenotype when confronted with a danger signal. Supporting the biological relevance of our results, a meta-analysis revealed that the genes showing the strongest up-regulation were also found up-regulated in acne. Of these genes, serum amyloid A 1/2 (SAA1/2) was confirmed to be a suitable protein marker for *in vivo* activated sebocytes, underlining their immune-competence, which is structurally defined within sebaceous glands of acne and rosacea skin samples. Altogether our findings demonstrate that sebocytes are not only positioned at the end point of inflammation but are actively involved in shaping the inflammatory response with putative diagnostic and therapeutic relevance.

## Introduction

To elucidate the complexity of acne pathogenesis, histological analysis complemented with genome wide expression profiling of lesional tissue samples has lately been performed [1–3]. Although the detected gene signatures could be paired with the inflammatory cell types accumulating around the sebaceous glands, such as macrophages/dendritic cells, neutrophils and Th17 lymphocytes [4], minor attempts were made to define the contribution of sebaceous glands to these inflammatory profiles. Such studies could answer whether sebocytes, not only contribute to the lipid barrier of the skin but also might be actively involved in shaping the inflammatory response [1, 5–14].

Sebocytes are able to respond to a wide repertoire of stimuli like pathogens, lipids and hormones reflecting the diversity of signals that could impact both the pathology as well as the possible treatment options in sebaceous gland-associated diseases, such as acne [15]. Of these factors, *Propionibacterium acnes (P*. *acnes)*, a Gram-positive bacterium present in the skin microbiome of acne prone areas [16], has gained a prime position [17–21], which by producing enzymes, such as lipases, proteinases and hyaluronidases could modify the composition of sebum [22]. Moreover, through the activation of TLR2, also expressed and regulated in sebocytes, *P*. *acnes* and its compounds could contribute to inflammation [8, 9, 23–27]. Supporting a role for other members of the microbiome, like Gram-negative bacteria, in the pathogenesis of acne, results showed that lipopolysaccharides (LPS; a Gram-negative bacteria-derived stimulus signaling through the TLR4 pathway) were also able to induce the expression of cytokines and chemokines, such as interleukin-6 (IL-6) and C-X-C Motif Chemokine Ligand 8 (CXCL-8) in sebocytes [10], which were detected also in *in vivo* sebaceous glands of acne samples [4, 7, 28]. In addition, antimicrobial peptides, such as human beta-defensin-2 [10], were also induced in response to LPS, whereas increased expression levels of cyclooxygenase-2 (COX-2), prostaglandin F2 alpha (PGF2a) and pro matrix metalloprotease-2 (proMMP-2) were also reported in LPS-treated hamster sebaceous glands [29]. These results indicate that TLR2 and 4 signaling pathways have a pivotal role in affecting sebocyte functions under pathological conditions and thus makes it relevant to characterize those in more details. Moreover, the question was raised whether the two TLR receptors mediate the same changes in sebocytes or have somewhat different effects, like in peripheral blood mononuclear cells (PBMCs) [9, 30–32].

In the present study, we aimed to provide answers at the level of gene expression regulation, gene expression profiling and pathway analysis of the altered genes by performing next generation high throughput sequencing in SZ95 sebocytes treated with specific synthetic TLR1/2 and TLR4 activators. By using meta-analysis of already available gene expression profile data from whole tissue samples of acne lesions [2], we also extended our work to define the magnitude of sebaceous gland contribution to inflammation. In addition, based on our gene expression data we aimed to identify a biomarker for activated sebaceous glands under *in vivo* conditions, which may be of diagnostic and therapeutic relevance.

## Materials and methods

### Cell culture and treatments

The SZ95 immortalized human sebaceous gland cell line [33] was cultured at 37°C in a humidified atmosphere containing 5% (v/v) CO_2_ in Sebomed Basal Medium® (Biochrom, Cambridge, UK) supplemented with 10% fetal bovine serum ([FBS], Biowest, Nuaillé, France), 1 mM CaCl_2_, 1% penicillin/streptomycin (Sigma-Aldrich, St. Louis, MO, USA) and 5 ng/ml epidermal growth factor ([EGF], Sigma-Aldrich). When reaching approximately 80% confluence, the medium was replaced with Sebomed Basal Medium® containing 0.5% FBS, 1 mM CaCl_2_, 1% penicillin/streptomycin, lacking EGF for 24 hours, followed by treatments with 1 μg/ml PAM3CSK4 (TLR1/2 activator; dissolved in sterile water; Cat. no.: TLRL PMS, InvivoGen, San Diego, CA, USA), 1 μg/ml LPS (TLR4 activator; derived from Escherichia coli; dissolved in sterile water; Cat. no.: L4391, Sigma-Aldrich) or 50 μM arachidonic acid (dissolved in 98% ethanol; Cat. no.: A3611, Sigma-Aldrich).

### ELISA measurements

In order to determine if the TLR1/2 and 4 activators (PAM3CSK4 and LPS) were efficient in the treated samples, levels of IL-6 and CXCL-8 were measured (S1 Fig) with DuoSet ELISA Development Kit (R&D Systems, Minneapolis, MN, USA) according to the manufacturers’ instructions. SZ95 sebocyte supernatants were collected at 24 hours after PAM3CSK4 and LPS treatments, and were aliquoted and stored at –20°C until further analyses. Samples were measured in triplicates for each cytokine. One-way ANOVA and Dunnett post-hoc test were used in the analyses of ELISA data.

### Determination of mRNA levels

SZ95 sebocytes were cultured in the presence of TLR1/2 and 4 ligands (PAM3CSK4 and LPS, respectively) or vehicle for 6 and 24 hours as described previously. Total RNA was isolated using TRI Reagent (MRC, Cincinnati, OH, USA) according to the manufacturer’s protocol and quantified by using NanoDrop 2000 (Thermo Fisher Scientific, Walthman, MA, USA).

For RNA sequencing (RNA-Seq) libraries were generated from 1 μg total RNA using TruSeq RNA Sample Preparation Kit (Illumina, San Diego, CA, USA) according to the manufacturer’s protocol. Briefly, poly-A tailed RNAs were purified by oligodT-conjugated magnetic beads and fragmented on 94°C for 8 min, then 1st strand cDNA was transcribed using random primers and SuperScript II reverse transcriptase (Life Technologies, Carlsbad, CA, USA). Following this step second strand cDNA was synthesized, the double-stranded cDNA was end-repaired, 3’ ends were adenylated then Illumina index adapters were ligated. After adapter ligation enrichment PCR was performed to amplify adapter ligated cDNA fragments. Fragment size distribution and molarity of libraries were checked on Agilent BioAnalyzer DNA1000 chip (Agilent Technologies, Santa Clara, CA, USA). Concentrations of RNA-Seq libraries were diluted to 10 nM and 5 libraries were pooled together before sequencing. Single read 50 bp sequencing run was performed on Illumina HiScan SQ instrument (Illumina). Each library pool was sequenced in one lane of sequencing flow cell, 16–18 million reads per sample was obtained.

### Analysis of RNA-Seq data

CASAVA software (Illumina) was used for pass filtering and demultiplexing process. Sequenced reads were aligned to Human Genome v19 using TopHat and Cufflinks algorithms and bam files were generated. Further statistical analyses were executed using NGS modul of GeneSpring 12.6 software (Agilent Technologies). Relative mRNA expression levels were calculated with DESeq algorithm. Aligned sequencing data have been deposited into the NCBI SRA database under accession no.: SRP126626.

### Western blot

Cells were washed in PBS and lysed in RIPA buffer containing a protease inhibitor mix (aprotinin, leupeptin, pepstatin, bestatin) (Sigma Aldrich). Proteins (20 μg) were separated by electrophoresis using appropriate polyacrylamide gel and transferred to nitrocellulose membrane (Bio-Rad, Hercules, CA, USA). After blocking, membranes were probed with anti-SAA1+SAA2 (Cat.no.: ab207445; Abcam, Cambridge, UK) antibody. The Ag–Ab complexes were labeled with appropriate HRP-conjugated secondary antibody (Bio-Rad) and visualized by WesternBright ECL HRP substrate (Advansta, CA, USA).

### Histological samples

Anonymized formalin-fixed and paraffin embedded (FFPE) sections of human skin from the tissue archive of the Department of Dermatology, University of Debrecen were acquired after the approval of the Regional and Institutional Ethics Committee, University of Debrecen. At least 5 different FFPE samples of each condition (healthy skin, papulopustular acne, papulopustular rosacea) were evaluated. Samples were collected from the back of acne, from the face of rosacea, and from matching skin areas of healthy individuals [34] who underwent surgery with histologically later confirmed benign melanocytic lesions.

### Immunohistochemistry

Slides from FFPE samples were deparaffinized and rehydrated. Endogenous peroxidase activity was blocked by treatment with 3% (v/v) H_2_O_2_ in distilled water for 15 min. Heat mediated antigen retrieval was performed with Tris-EDTA buffer (Tris Base, 1mM EDTA solution, 0.05% Tween 20, pH 9). Nonspecific binding was blocked with 1% bovine serum albumin (BSA) in PBS for 30 min at room temperature. Anti-SAA1+SAA2 (Cat. no.: ab 207445; Abcam) antibody was diluted 1:200 in PBS and sections were incubated for 1 h at room temperature in humidity chambers. For negative controls, the appropriate non-immune control sera (rabbit IgG; Vector Laboratories, Burlingame, CA, USA) were used instead of the primary antibody. HRP-conjugated secondary antibody was used in accordance with the manufacturer’s instructions (SuperSensitive One-step Polymer-HRP Detection System, BioGenex Laboratories, Fremont, CA, USA). Immunoreaction was visualized by Vector VIP Kit (Vector Labs.) Sections were counterstained with methylene green. Images were acquired with a Leica DM2000 LED microscope (Leica Microsystems, Wetzlar, Germany).

### Oil Red O staining

SZ95 sebocytes were cultured on coverslips in the presence of TLR1/2 and 4 ligands (PAM3CSK4 and LPS, respectively), arachidonic acid or vehicle for 24 and 48 hours as described previously. Cells were fixed in 4% paraformaldehyde (Sigma-Aldrich) for 10 min. Coverslips were placed in 100% propylene glycol (Amresco, Solon, OH, USA) for 1 min, followed by a rinse with distilled water. Cells were stained with 0.7% Oil Red O (Sigma-Aldrich) solution for 7 minutes then rinsed with 85% propylene glycol solutions. Nuclei were counterstained with hematoxylin and slides were covered using Mount Quick Aqueous mounting medium (Bio Optica, Milano, Italy).

## Results

### Global transcriptome analysis reveals a permanent response of SZ95 sebocytes to TLR1/2 and 4 activators

**Fig 1:**
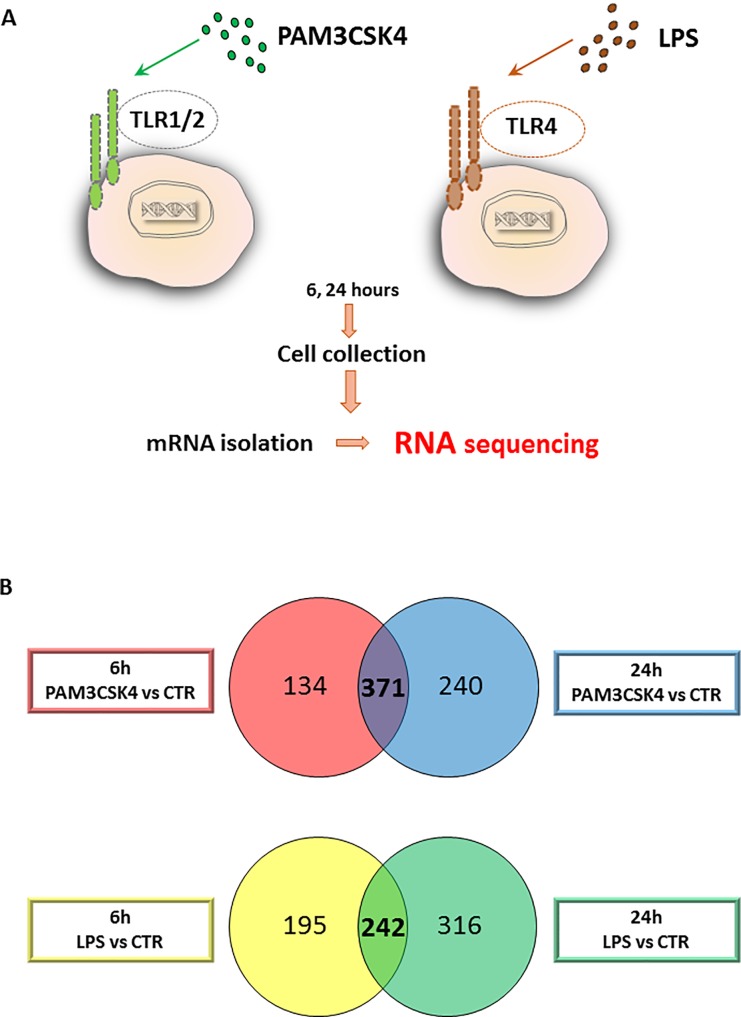
Sebocytes respond to TLR 1/2 and 4 activation. (A) Experimental setting to detect the immediate and the later changes at the level of gene expression regulation in sebocytes upon TLR1/2 and TLR4 activation. SZ95 sebocytes were cultured in the presence of PAM3CSK4 and LPS to selectively and specifically activate the TLR1/2 and TLR4 pathways. Samples for RNAseq measurements were harvested at 6 and 24 hours after treatment, processed and measured as described in Materials and Methods. (B) Number of genes regulated by PAM3CSK4 (TLR1/2) or LPS (TLR4) in SZ95 sebocytes at early and late time points were identified and compared. The results are visualized as a Venn diagram. The regulated mRNAs were categorized as early and late based on their significantly altered expression levels at 6 hours (red circle for PAM3CSK4 and yellow for LPS) and 24 hours (blue circle for PAM3CSK4 and green for LPS) retrospectively when compared to untreated cells. Note the large number of genes that are regulated already at 6 hours and the high number of overlapping genes in the 6 and the 24 hour samples in response to both treatments.

To identify the transcriptional changes in sebocytes upon activation of TLR1/2 and TLR4 pathways, we treated SZ95 sebocytes with the specific, selective activators PAM3CSK4 (TLR1/2 pathway activator) and LPS (TLR4 pathway activator). By choosing a 6-hour time point for our studies we focused on the initial changes, while the 24-hour time point allowed us to address the ability of sebocytes to maintain their altered gene expression profile and thus their commitment towards inflammation (Fig 1A). Identifying the transcriptomes that reached a significance in all biological triplicates at a given time point clearly fulfilled our criteria to define genes with a statistically relevant altered expression.

As a first step, we focused on the changes at 6 hours and found a significant expression change of approximately 500 genes in response to the administered TLR1/2 and TLR4 activators (505 in response to PAM3CSK4 and 437 in response to LPS). Next, we determined the changes at 24 hours and found a further increase in the number of the differentially expressed genes (611 in response to PAM3CSK4 and 558 in response to LPS). Interestingly, 43.3% (in case of PAM3CSK4) and 60% (in case of LPS) of the genes from the 6-hour time point were still having significantly changed expression levels at 24 hours (Fig 1B).

To assess the changes that were found at 6 but not at 24 hours we performed functional clustering of the relevant genes also presented in a heat map form (S2A Fig). Interestingly, we could not confirm a clear signature or function for these genes (S2B Fig) that could mark a biologically relevant temporary effect of the applied stimuli.

These results altogether showed that the response of sebocytes to the used TLR activators is not a transient state but induces long term changes in the genetic programs.

### TLR1/2 and TLR4 signaling induces similar changes in the gene expression profile of SZ95 sebocytes

**Fig 2:**
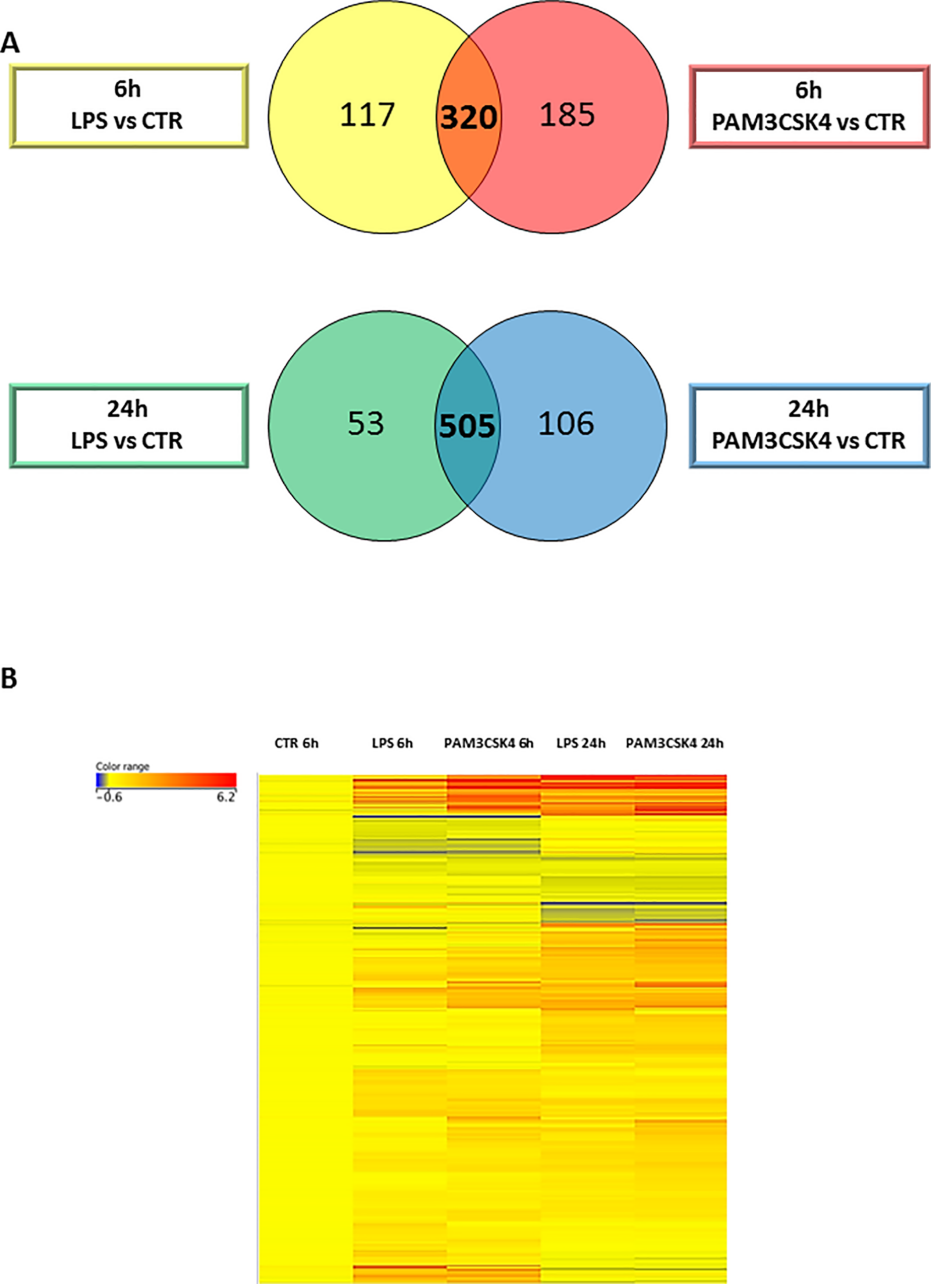
TLR1/2 and TLR4 pathways induce similar changes in the gene expression profile of SZ95 sebocytes. (A)Venn diagram visualizing genes that are regulated both by PAM3CSK4 and LPS in SZ95 sebocytes at 6 (orange) and 24 hours (dark blue). Note the large number of genes that are regulated by both stimuli at 6 hours and an even greater number at 24 hours. (B) Differentially expressed genes in untreated, LPS or PAM3CSK4 treated SZ95 sebocytes at 6 and 24 hours as observed by our RNAseq analysis shown as a heat map. Color intensities reflect the ratios of signal intensities as shown. Note that the detected changes were due to a sustained up-regulation in the vast majority of genes, while no genes with a significant down-regulation at both time-points were detected.

To detect how close the two signaling pathways are in their effects on differential gene expression profiles, we compared the gene lists of both conditions of TLR-activated sebocytes from the relevant time points. We found that the differentially expressed genes showed a prominent overlap (320 out of 437 [LPS] and 505 [PAM3CSK4]) at 6 hours, while at 24 hours the gene expression profile was almost identical (over 80% in case of PAM3CSK4 and 90% in case of LPS) (Fig 2A).

Hierarchical clustering of the genes that were expressed and changed between any conditions in unstimulated, TLR1/2- and TLR4-activated sebocytes exhibited changes mostly due to up-regulation, as only approximately 20% of the genes were down-regulated. This prominent induction in the number of regulated genes suggests that sebocytes respond to the activation of the TLR 1/2 and TLR4 pathways by adding a new profile to their transcriptome rather than losing the one from their untreated state. This was further supported by pathway analysis of the down-regulated genes that could not sort them into any functional category.

The generated heat map also showed that of the genes reaching the level of significance at 24 but not at 6 hours, a very high number were already showing tendency of up-regulation at 6 hours, supporting that most of the changes observed at 24 hours occurred directly through the applied TLR stimuli and that sebocytes were already committed at this early time point towards their new profile (Fig 2B).

### Functional clustering of the TLR1/2 and TLR4 responding genes in SZ95 sebocytes reveals an immediate change in immune response and only a later one in lipid metabolism

To assess the biological response and functions that TLR1/2- and TLR4-activated sebocytes could gain at the level of gene expression, we extended our studies with gene clustering and pathway analysis of the altered genes at 6 and at 24 hours. Notably, as it was expected from the previously detailed similarities in the genes regulated by TLR1/2 and TLR4 pathways, the functional analysis also resulted in the same clusters in both treatments. This was confirmed by using two independent softwares (Panther and Cytoscape pathway analysis programs).

Analysis of the altered genes from the 6-hour time point revealed that the most significant changes were related to genes involved in immune system/defense processes underpinning that TLR 1/2 and TLR4 pathways are also fully active in sebocytes and are a potential link between the inflammatory environment and the gene expression profile also in this cell type. The gene clustering provided many interesting clusters, as well as novel ones, to sebocyte research: such as a possible involvement in wound healing, regulation of cell proliferation, chemotaxis and a more complex cytokine and chemokine production than what was previously known besides the widely used detection of IL-6 and CXCL-8.

The analysis of the 24-hour samples found the same clusters as at 6 hours, which was in line with the gene expression data showing that the changes at 6 hours were also consistent in the 24-hour samples. However, the cluster of cholesterol and steroid metabolic processes, which represents the key function of sebocytes to produce lipids, was only affected at the 24-hour time point (Table 1) with a significant up-regulation in the expression of the cluster forming genes but with the exception of *ABCG1* that was down-regulated (S1 Table). Importantly, this also provides a possible explanation for our observations that no change was detected in the lipid body formation of TLR1/2- and TLR4-activated sebocytes, which is the morphological hallmark for an altered lipid metabolism (S3 Fig).

**Table 1:** Clustering of the altered genes in response to TLR1/2 or TLR4 activation in SZ95 sebocytes. Genes that were regulated both by PAM3CSK4 and LPS were functionally categorized using the Cytoscape classification system. The clusters clearly defined an immunocompetence for the activated sebocytes and pointed on so far unrevealed functions such as a possible involvement in wounding and leukocyte migration. Note that in black are the genes/clusters present in both the early (6 hours) as well as in the late responder (24 hours) group, while the genes in bold falling to the functional category of cholesterol metabolic process were only detected at 24 hours.

GO-ID	p-value	corr p-value	Description	Genes in test set
2376	1,46E-38	4,38E-35	immune system process	CXCL6|CSF3|CIITA|CSF2|CSF1|SECTM1|CXCL1|CXCL3|TNF|CXCL2| CTSS|IFI44L|CXCL5|ICAM1|OASL|IFIH1|C4B|C4A|KYNU|DHX58|C1RL|TRIM25|JUNB|IKBKE|CD34|ICOSLG|TRIM22|SERPINB4|EDN1|IL4R| IL1R1|DDX58|FST|WNT5A|TAP2|TAP1|TNFRSF1B|HLA-F|HLA-G|MMP9|TAPBP|OAS1|OAS2|IL1B|IRF1|OAS3|LCP1|LTB|S100A|CFB|LTF|PTGER4|SELPLG|C1S|C1R|EBI3|NOD2|FYB|RELB|C3|SBNO2|EBP|IFI16|NFIL3|IRAK2|CCL5|UBD|CCL2|APOL1|GBP2|TNFSF18|IL32| CD74|TGFB2|VCAM1|CCL20|INHBA|SOD2|PML|NFKB2|BST2|NFKBI| CXCL10|CXCL11|IL6|VNN1|BCL6|IL8|BCL3|LCN2|SAA1|TNFSF9|SAA|PTX3|IL7R
6955	4,97E-32	7,47E-29	immune response	CXCL6|CSF3|CIITA|CSF2|SECTM1|CXCL1|CXCL3|TNF|CXCL2|CTSS| IFI44L|CXCL5|ICAM1|OASL|IFIH1|C4B|C4A|KYNU|DHX58|C1RL| TRIM25|IKBKE|TRIM22|SERPINB4|IL4R|IL1R1|DDX58|WNT5A| TNFRSF1B|HLA-F|HLA-G|TAPBP|OAS1|OAS2|IL1B|OAS3|LCP1|LTB|CFB|LTF|PTGER4|C1S| C1R|EBI3|NOD2|FYB|RELB|C3|SBNO2|NFIL3|CCL5|UBD|CCL2| APOL1|GBP2|TNFSF18|IL32|CD74|CCL20|NFKB2|BST2|CXCL10| CXCL11|IL6|VNN1|IL8|BCL3|LCN2|TNFSF9|PTX3|IL7R
6952	3,98E-31	3,99E-28	defense response	CXCL6|CIITA|SERPINE1|CXCL1|CXCL3|TNF|CXCL2|IFIH1|C4B|C4A| KYNU|DHX58|C1RL|NFKBIZ|BDKRB2|OLR1|TRIM25|AOX1|ICOSLG| IL1R1|DDX58|WNT5A|PLA2G4C|TAP1|F3|HLA-G|ADORA2A|ELF3|IL1B|LAP3|S100A9|CFB|S100A8|IDO1|LTF|SAA3P|C1S|C1R|SAA4|NOD2|C3|IRAK2|CCL5|UBD|CCL2|APOL1|APOL3|IL32|CD74|VCAM1|KLRC2|CCL20|MX2|STAT3|MX1|INHBA|NFKB1|ASS1|FOSL1|CXCL10|CXCL11|IL6|VNN1|IL8|TNIP1|BCL3|LCN2|SAA1|SAA2|PTX3
9611	1,60E-24	9,60E-22	response to wounding	NRP1|CXCL6|CIITA|SERPINE1|ADM|CXCL1|CXCL3|TNF|CXCL2|C4B| THBD|C4A|PLAU|C1RL|NFKBIZ|BDKRB2|OLR1|AOX1|IFNGR1| WNT5A|PLA2G4C|F3|EREG|PLSCR1|ADORA2A|LOX|ELF3|IL1B|LAP3|S100A9|CFB|S100A8|IDO1|SAA3P|C1S|C1R|SAA4|TFPI|C3|IRAK2| CCL5|CCL2|APOL3|TGFB2|VCAM1|CCL20|STAT3|SOD2|NFKB1|ASS1|CXCL10|CXCL11|IL6|VNN1|IL8|SAA1|SAA2|PTX3
6954	3,27E-24	1,64E-21	inflammatory response	SAA3P|CXCL6|CIITA|C1S|C1R|SAA4|CXCL1|CXCL3|TNF|CXCL2|C3| C4B|C4A|IRAK2|CCL5|C1RL|NFKBIZ|BDKRB2|OLR1|CCL2|AOX1| APOL3|VCAM1|CCL20|STAT3|PLA2G4C|F3|NFKB1|ASS1|CXCL10| CXCL11|IL6|VNN1|ADORA2A|IL8|ELF3|IL1B|SAA1|SAA2|PTX3|LAP3|S100A9|CFB|S100A8|IDO1
6950	1,34E-22	5,74E-20	response to stress	SERPINE1|TNF|C4B|C4A|PLAU|DHX58|C1RL|TRIM25|ICOSLG|IL1R1|WNT5A|DIO2|TAP1|HLA-G|EREG|LOX|LAP3|TRIB1|S100A9|CFB|S100A8|IDO1|INSIG1|ADRB2|C3|SOCS3|IRAK2|UBD|JUN|TGFB2|VCAM1|KLRC2|DHCR24|INHBA| NFKB1|FOSL1|CXCL10|CXCL11|IL6|VNN1|BCL6|IL8|BCL3|MAFF| LCN2|NRP1|CXCL6|CIITA|PCSK9|ADM|CXCL1|CXCL3|CXCL2|HK2| IFIH1|THBD|DUSP10|UCN2|KYNU|NFKBIZ|BDKRB2|OLR1|AOX1| IKBKE|EDN1|IFNGR1|DDX58|PLA2G4C|F3|PLSCR1|ADORA2A|ELF3| IL1B|ALDOC|SGK1|LTF|SAA3P|C1S|C1R|SAA4|NOD2|PTGS2|TFPI| IFI16|CCL5|CCL2|APOL1|APOL3|IL32|ARNT2|CD74|CCL20|STAT1| MX2|STAT3|MX1|SOD2|AGT|ASS1|PML|TNIP1|SAA1|SAA2|PTX3
42127	5,99E-17	1,20E-14	regulation of cell proliferation	NRP1|CSF3|IFITM1|CSF2|CSF1|SERPINE1|PTPRO|ADM|CXCL1|TNF| CXCL5|CDH5|PLAU|MYC|NAMPT|BDKRB2|ICOSLG|TGM2|FGFBP1| EDN1|SP110|IGFBP3|WNT5A|SSTR1|F3|RUNX3|EREG|ADORA2A| IL1B|MVD|TRIB1|IDO1|EBI3|ADRB2|PTGS2|CCL2|ARNT2|CD74|JUN|TGFB2|VCAM1|STAT1|TNFRSF9|IL34|RARRES1|VEGFC|DHCR24| INHBA|SOD2|AGT|PML|FOSL2|FOSL1| NFKBIA|CXCL10|IL6|BCL6|FAP|IL8|TNFSF9|DHCR7
**8203**	**3,55E-16**	**6,67E-14**	**cholesterol metabolic process**	**IDI1|FDPS|MVK|HMGCS1|INSIG1|CYP51A1|PCSK9|DHCR24| HMGCR|LSS|ACAT2|SQLE|NSDHL|EBP|FAP|MVD|DHCR7|APOL1| LDLR|APOL3|FDFT1**
2526	6,81E-13	7,58E-11	acute inflammatory response	SAA3P|VCAM1|C1S|C1R|STAT3|SAA4|F3|ASS1|C3|C4B|C4A|IL6| VNN1|C1RL|SAA1|SAA2|CFB|APOL3
50900	3,99E-12	3,99E-10	leukocyte migration	TGFB2|SELPLG|VCAM1|CXCL3|TNF|ICAM1|IL6|IL8|CCL5|IL1B|SAA1|CCL2|SAA2|S100A9|CD34

Altogether, these findings were surprising, since a cell type with a primary role to produce lipids was not promptly changing its lipid profile on the level of gene expression or lipid body formation. On the other hand, the experiments delivered interesting data on how rapidly sebocytes could add the inflammatory status to their profile and accommodate to a new environment.

### Meta-analysis of gene expression profiles of acne samples and TLR1/2- and TLR4-activated SZ95 sebocytes suggests a possible contribution of sebocytes to disease associated inflammation

The available microarrays from whole tissue biopsies of acne samples (GSE53795) [2], histological analysis and *in vitro* experiments all suggested a role for the TLR1/2 and TLR4 pathways [9, 23] in the pathogenesis of acne. However, no cell type-specific association with disease development and inflammation was addressed in response to these stimuli. Therefore, as a next step, a meta-analysis was performed using available gene expression data of acne whole tissue samples [2] and the list of the up-regulated genes in TLR-stimulated SZ95 sebocytes to determine possible genes and pathways with which sebocytes could integrate into the TLR2/TLR4-acne cascade.

**Fig 3:**
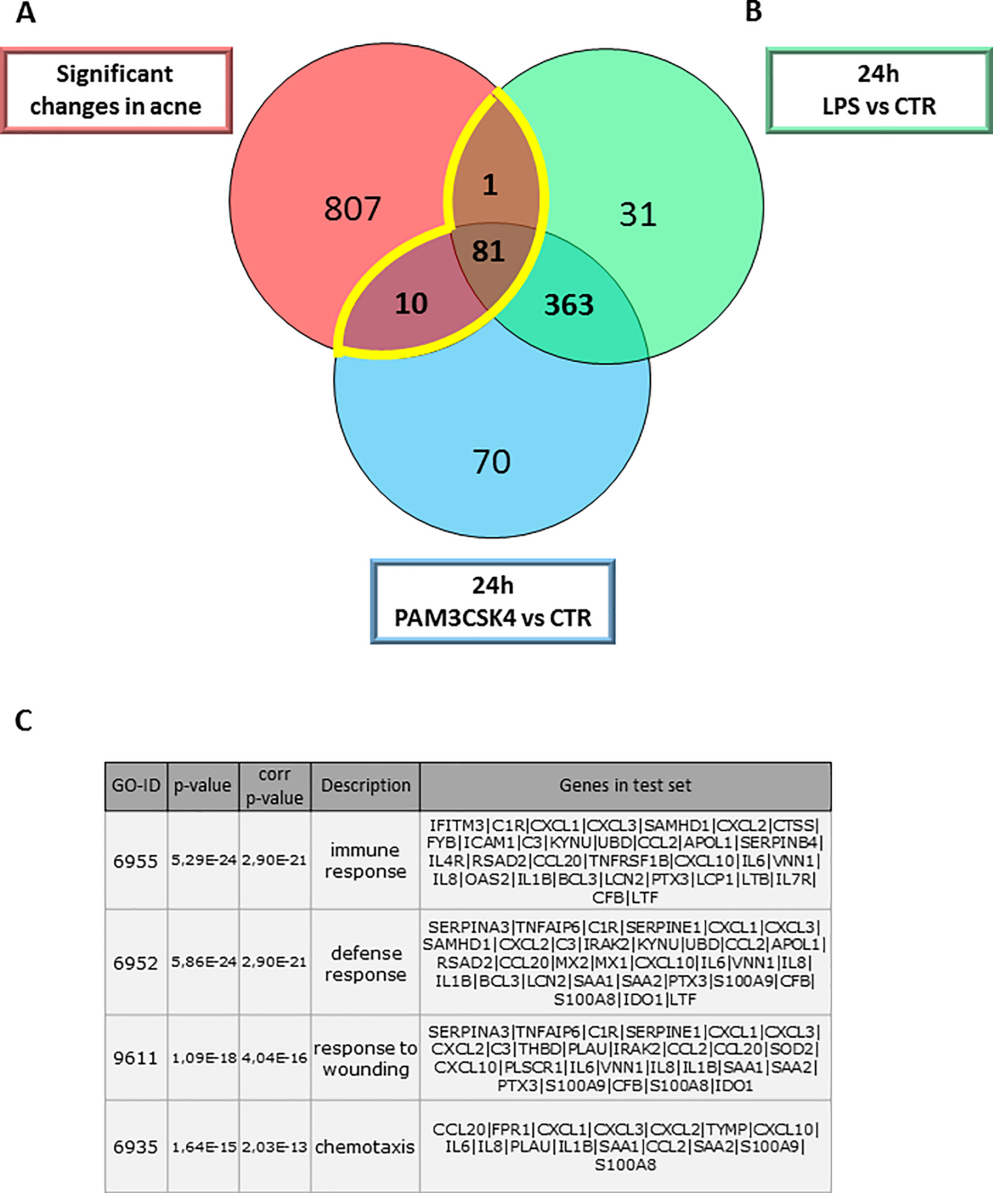
Meta-analysis using gene expression profiles from acne samples, TLR1/2- and TLR4-activated SZ95 sebocytes reveals a possible contribution of sebocytes to the inflammatory environment in acne. (A) Genes significantly up-regulated in acne samples (red circle) when compared to control samples and genes up-regulated in LPS (green circle) or PAM3CSK4 (blue circles) treated SZ95 sebocytes when compared to untreated cells at 24 hours are visualized as a Venn diagram. Gene expression data of acne samples were obtained from available gene expression profiles (2). (B) Overlapping genes presented in a heat map were functionally categorized using the Cytoscape classification system. (C) Biological process analysis by Cytoscape Analysis confirmed that sebocytes are possible candidates for contributing to the inflammatory environment in acne samples.

Using a Venn diagram to visualize the up-regulated genes in the acne samples compared to healthy ones and in the TLR1/2- and TLR4-stimulated SZ95 sebocytes at 24 hours, a possible contribution of sebocytes with 92 of the 900 significantly altered transcripts in acne could be detected (Fig 3B).

To assess how sebocytes may contribute to acne pathogenesis, further analysis was performed using only the overlapping 92 genes (Fig 3A). The genes were grouped into functions such as immune response, defense response, response to wounding, response to stress and chemotaxis (Fig 3C).

### Serum amyloid A 1/2 marks activated sebocytes and reveals structural differences in the immune-competence of sebaceous glands

**Fig 4:**
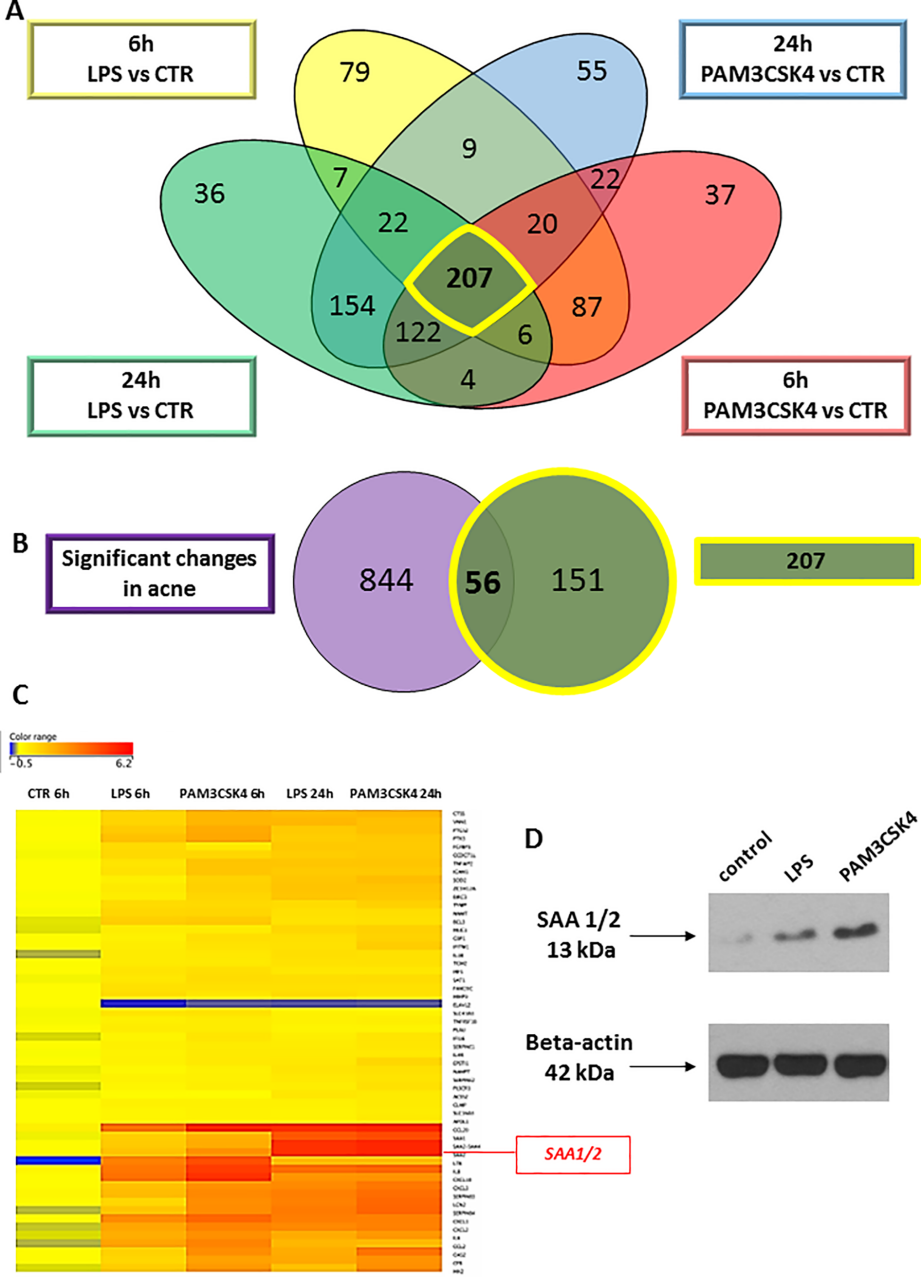
Identifying Serum amyloid A 1/2 as a marker for inflamed sebocytes. (A)Venn diagram visualizing the number of genes showing significantly different expression levels between treatment conditions (LPS and PAM3CSK4 treated SZ95 sebocytes at 6 and 24 hours when compared to untreated SZ95 sebocytes) as observed by our RNAseq analysis. Note that 207 genes were identified as differentially regulated in any of the examined conditions and time points, marked by yellow. (B) Venn diagram visualizing the overlap of the 207 genes and the differentially regulated genes in acne samples from available gene expression profiles (2). Note that 56 out of the 207 genes were significantly regulated also in acne samples. (C) Hierarchical clustering of the 56 genes that are differentially expressed in any of the conditions of LPS and PAM3CSK4 treated SZ95 sebocytes when compared to untreated cells both at 6 and at 24 hours and are also significantly altered in acne samples when compared to control ones as observed in the gene expression profiles of acne samples from the available work of Kelhala HL et al. (NCBI GEO accession number: GSE5379) (2). Out of the 56 genes presented on the heat map, Serum amyloid A 1/2 is highlighted, fulfilling our criteria (up-regulated in acne samples as well as by both treatments, its expression levels increased from 6 to 24 hours, detectable also at the level of protein) to define a possible marker for inflamed sebocytes. (D) Western blot analysis of SAA1/2 in control, LPS and PAM3CSK4 treated SZ95 sebocytes 24 h after treatment.

Next, we aimed to analyze and describe markers for TLR 1/2- and TLR4-mediated signaling in sebocytes. The candidate genes, which showed the most robust induction upon PAM3CSK4 and LPS treatments, compared to non-stimulated sebocytes at both 6 and 24 hours were selected from the RNAseq results. The list of 207 genes were then visualized in a Venn diagram with the genes that were differentially regulated in acne samples when compared with healthy control (Fig 4A). The overlapping 56 genes were subjected to hierarchical clustering to identify the ones with the highest change of expression in all conditions and to choose the ones whose expression showed a further increase from 6 to 24 hours in the stimulated SZ95 sebocytes (Fig 4B). As a result Serum Amyloid A 1/2 *(SAA1/2)* was identified to be suitable marker to identify activated sebocytes at the level of gene expression (Fig 4C). Furthermore, correlating with the mRNA data, the protein levels of SAA1/2 were also induced in TLR1/2 and 4 activated SZ95 sebocytes as revealed by Western blotting (Fig 4D).

**Fig 5:**
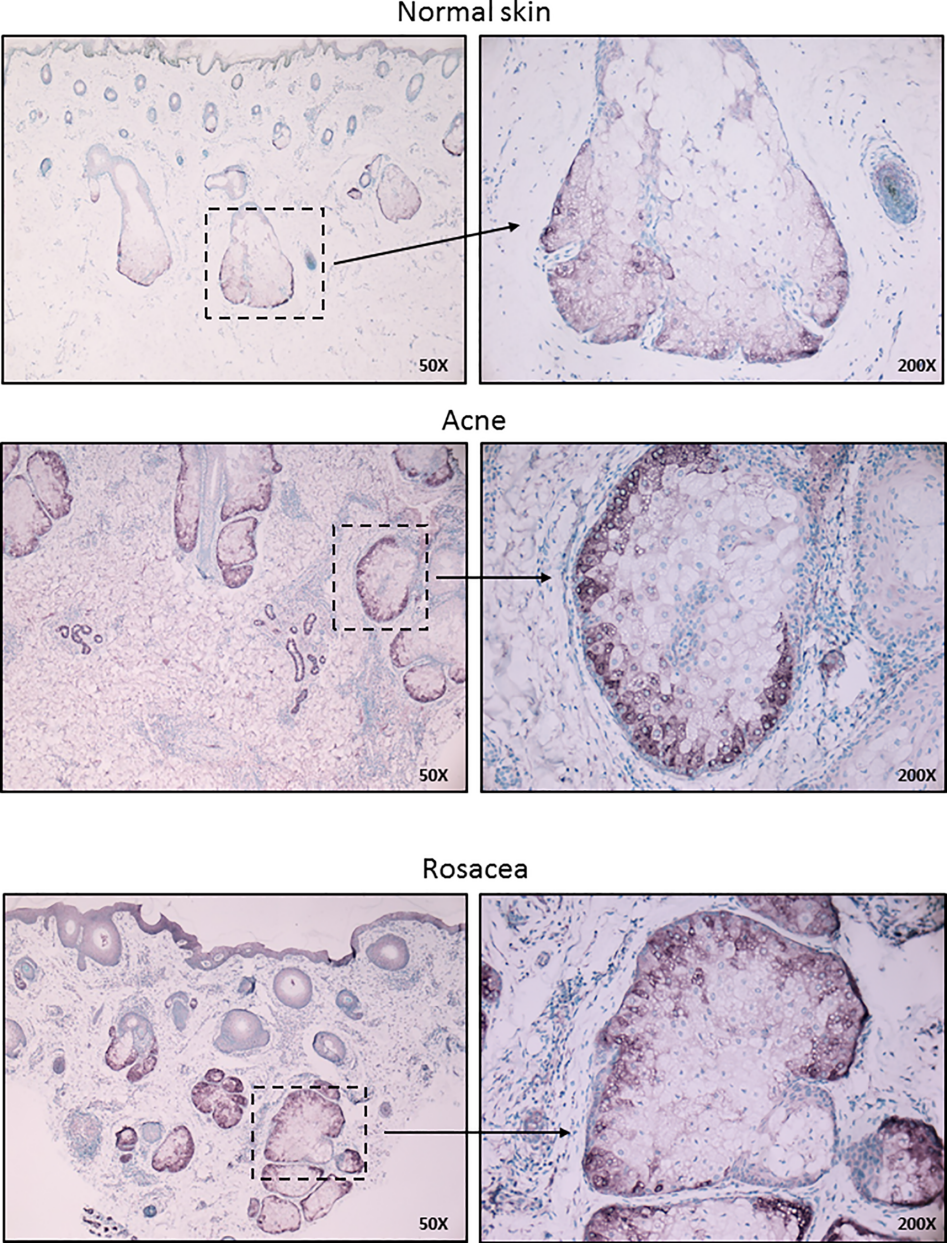
Immunohistochemical detection of Serum Amyloid A 1/2 marks activated sebocytes and reveals a structurally defined immune-competence in sebaceous glands. Immunohistochemical detection of SAA1/2 in human sebaceous glands in control skin as well as in papulopustular acne and papulopustular rosacea. FFPE tissue samples were stained with rabbit monoclonal SAA1/2 antibody as described in the Materials and Methods. Note that besides the increased staining intensities observed in papulopustular acne and papulopustular rosacea samples, SAA1/2 positivity had a characteristic distribution within the sebaceous glands, localizing exclusively to the basal cell layers. Images are representative of at least 5 samples from each disease and each staining. Staining intensities were independent of age and gender. Sections were counterstained with methylene green. Original magnification: 50X, 200X.

To assess if the detection of SAA1/2 could also be applied and therefore be routinely used to mark activated sebaceous glands at the protein level in histological specimens, we performed immunohistochemistry using specific antibodies on normal as well as pathological skin samples such as papulopustular acne and papulopustular rosacea. SAA1/2 was successfully detected, with prominent differences in the staining intensities of sebaceous glands in papulopustular acne and papulopustular rosacea samples when compared with healthy skin. Importantly, SAA1/2 positivity had a characteristic distribution within the sebaceous glands in both diseases, localizing exclusively at the basal cell layers, revealing a well-defined functional architecture of sebaceous glands regarding its immune-competence (Fig 5).

## Discussion

To explore the possible functions for sebocytes under inflammatory conditions, we applied a system-based approach of whole genome sequencing of SZ95 sebocytes, the best characterized sebocyte line [33], treated with specific and selective TLR1/2 and TLR4 activators. This unbiased strategy extends beyond the limits of previous studies that have only focused on a selected set of proteins [35, 36]. With strict criteria in our statistical analysis and also applying profiles of available gene expression data of whole tissue samples of acne lesions [2], we used all available tools to detect the differentially expressed genes in order to gain a step-by-step view and follow-up on the accommodation of sebocytes to the TLR1/2 and TLR4 activators.

One of the most interesting findings in our study was that the TLR1/2 and TLR4 pathways induced a similar change in the gene expression profile of the SZ95 sebocytes and that this change was already present to a great extent at 6 hours. Pathway analysis of the altered genes provided the same clusters regardless of the applied activator, which served to corroborate our findings and further indicate so far unrevealed functionality for sebocytes under inflammatory conditions. Importantly, most of these clusters were also present in the meta-analysis of acne whole tissue samples suggesting that sebocytes may contribute to disease-specific inflammatory signatures as well. These findings, therefore, extend far beyond the complexity of the genetic programs that are inducible in sebocytes and suggest that these cells may be active players in the pathogenesis of acne.

From the detected clusters, the one showing the most significant changes and also including the highest number of genes was related to inflammation. Although this change was expected based on previous results, still it was surprising to see that many of the cluster-forming genes (*C3*, *SERPINA3*, *IL6*, *VNN1*, *C1R*, *SAA1*, *SAA2* and *CFB*) encode for proteins that are known serum markers for severe systemic inflammation in various diseases. Moreover, as revealed by a number of genes (*SERPINA3*, *TNFAIP6*, *C1R*, *SERPINE1*, *C3*, *THBD*, *PLAU*, *IRAK2*, *SOD2*, *PLSCR1*, *VNN1*, *SAA1*, *SAA2*, *PTX3*, *S100A9*, *S100A8*, *CFB*, and *IDO1*), sebocytes via their products might also contribute to wound healing which is also a novel field yet to be characterized and could bring us closer to understand the background of acne-associated scarring. Another interesting outcome of our analysis was the high number of cytokines induced at the level of gene expression, which besides *IL1β*, *IL6* and *CXCL8*, have not yet been characterized in sebocyte research (*CXCL1*, *CXCL2*, *CXCL3*, *CXCL10*, *CCL2* and *CCL20*). Further clustering of these genes clearly showed that the wide repertoire of cytokines might have an effect on chemotaxis and thus have a role in initiating inflammation. This has been addressed in our recently published work, where after showing that TLR 2 and 4 activation of SZ95 sebocytes led to an induction also in the levels of the measured proteins (CXCL-8, CXCL-10 CCL-5, IL-6, G-CSF and VEGF) from the supernatants of cell cultures, we demonstrated a CXCL-8-dependent chemotactic effect of sebocytes on neutrophils, monocytes and T cells [37].

Lipid metabolism, the primary function of sebocytes, is also greatly altered both in quantity as well as in quality under inflammation as revealed with the analysis of sebum from patients with acne as well as other diseases [21, 38–40]. Therefore, one of the most intriguing data from our study was to see how the examined TLR pathways could influence lipid metabolism at the level of gene expression. Unexpectedly, of the genes that encode proteins related to lipid metabolism, a significant change and thus representation in the pathway clustering was only detected in the 24-hour time point samples. However, at that time point, a great number of genes from the steroid biosynthetic processes were altered (*IDI1*, *FDPS*. *MVK*. *HMGCS1*, *INSIG1*, *CYP51A1*, *PCSK9*, *DHCR24*, *HMGCR*, *LSS*, *ACAT2*, *SQLE*, *NSDHL*, *EBP*, *FAP*, *MVD*, *DHCR7*, *APOL1*, *LDLR*, *APOL3*, *FDFT1* and *ABCG1*). Concluding on the enzymatic activity based on gene expression result would be oversimplified, still these data provide a strong piece of evidence, that sebocytes are indeed able to link inflammation to lipid metabolism in which the TLR1/2 and TLR4 pathways could have a pivotal trigger effect [21, 40].

Altogether, the detected changes in the induced genes and their clustering allowed two major conclusions: i., sebocytes are able to rapidly gain an immune-competent status in response either to TLR1/2 and or TLR4 activation. The clustering data fully support that sebocytes are not simply lipid-metabolizing cells, but should also be challenged as inflammatory modulators in a wide spectrum of dermatological diseases beyond acne. ii., The detected inflammatory responses were neither TLR1/2- nor TLR4-specific, in other words sebocytes are not able to sense and respond selectively to the two stimuli of different origin. This finding gains its importance when put into the current concept of pathogen-related inflammation of acne lesions suggesting that various stimuli, in and around the pilosebaceous unit capable of activating the TLR2 and TLR4 pathways, might all be able to modulate the inflammatory status of the sebaceous glands. Therefore, it is more likely that *P*. *acnes* has its specific role in acne pathogenesis not (only) via the TLR2 pathway but with its various products that could interact with the inflammatory pathways, which is definitely a challenging field for future studies [41, 42].

Another important outcome of our study was the identification of SAA1/2 as a marker protein for activated sebocytes, which might not only be a marker but could have a role as well in various disease settings. SAA1/2 is predominantly produced by the liver and by adipocytes, in strong association with the body mass [43], while its elevated serum concentration marks the acute phase of inflammation. Moreover, SAA1 has been found to increase also in keratinocytes in response to TLR2 activation, which was further augmented when glucocorticoids were used in combination, suggesting a possible contribution of SAA to (steroid-induced) acne [44]. Importantly in our *in vivo* studies, sebocytes also stained strongly positive for SAA1/2 suggesting that they may as well contribute to the levels of SAA1/2 in the local tissue environment and perhaps even in the serum, which calls for further studies to assess it in acne patients. Besides being a strong chemoattractant, SAAs also transport cholesterol that immediately puts forward the question whether sebocyte-produced lipids may contribute not just to sebum but might also make their way to the circulation via SAA. The finding that SAA has also been identified as a danger signal that triggers activation and IL-1β secretion, a key cytokine in acne pathogenesis, sets altogether the basis for intriguing speculations on sebocyte derived SAA to be tested as a possible therapeutic target [45]. Importantly, our finding that sebaceous glands were positive not just in acne samples, but also in rosacea supports, that sebaceous glands could be activated in other inflammatory conditions besides acne, with a pathomechanism and role yet to be defined (15). Of further interest is the finding that SAA1/2 immunoreactivity was restricted to the basal cells, similarly to what Alestas et al. have observed in the case of IL6 [7], suggesting a functional compartmentalization, which deserves further exploration. This finding gains its importance when answering the question what protects sebocytes from their own secreted lipids, of which many are known inflammatory activators [46]. Based on our results we put forward the hypothesis that while the basal cell layer is a potential immune responder part of the sebaceous glands, with the maturation, the upper layers lose their immune-potential thus gaining a possible protection against lipids. To identify the underlying changes is definitely a challenging field involving epigenetic as well as protein and signaling pathway studies.

In summary, our findings confirm that human sebocytes, with a primary role to metabolize lipids, are able to rapidly acquire an immune-competent status when exposed to danger signals and this function is structurally defined within the sebaceous glands. Thus sebocytes may not just be at the end-point of inflammation but also contribute to disease development [7, 10, 11, 21, 28, 47–54]. Our data also put forward the need to revisit the role for the *P*. *acnes–*TLR2 pathway and to reveal the TLR2 independent effects of *P*. *acnes* as well, which could provide clues to understand the exclusive pathogenic features of *P*. *acnes* in the pathogenesis of acne. Moreover, we suggest that the different mechanisms that could regulate the levels of the TLR2 and 4 receptors, or interact with their signaling pathways [24, 25, 55] should be included more into the concepts of acne pathogenesis and treatment options. By identifying SAA1/2 as marker for immune-competent sebocytes, we also provided a useful research tool to extend our knowledge on diseases which could be associated with sebaceous glands, and provide a possible target for anti-inflammatory therapeutic interventions to treat sebaceous gland-associated diseases.

## Supporting information

S1 Fig Levels of IL-6 and CXCL-8 in the supernatants of TLR1/2 (PAM3CSK4) and TLR4 (LPS) activated SZ95 sebocytes used for RNAseq analyses. Protein levels of IL-6 and CXCL-8 were measured by ELISA as described in Materials and Methods. One-way ANOVA and Dunnett post-hoc test were used in the data analyses (n = 3); * = p < 0.05, ** = p <0.01, *** = p <0.0001.
(TIF)

S2 Fig Heat map display of the genes with differential expression levels only at 6 hours. (TIF)

S3 Fig Oil Red O staining of lipid bodies in TLR1/2- (PAM3CSK4), TLR4- (LPS) activated or arachidonic acid (AA) treated SZ95 sebocytes. Oil Red O staining revealed no changes in the lipid body formation of TLR1/2- and TLR4-activated sebocytes neither at 24-hour nor at 48-hour time points. Arachidonic acid (AA) treatment inducing lipid body formation both in numbers and size was used as a positive control. At least three independent samples were stained per each treatment.
(TIF)

S1 Table Genes involved in lipid metabolism with altered expression levels at 24 hours. Note the significant up-regulation in the expression levels of the cluster forming genes with the exception of ABCG1.
(DOCX)
