# Supplementary figures and images for: Genome wide analysis of TLR1/2- and TLR4-activated SZ95 sebocytes reveals a complex immune-competence and identifies serum amyloid A as a marker for activated sebaceous glands
Source: PLoS One. 2018 Jun 21;13(6):e0198323. doi: 10.1371/journal.pone.0198323 (PMC6013244; doi:10.1371/journal.pone.0198323)

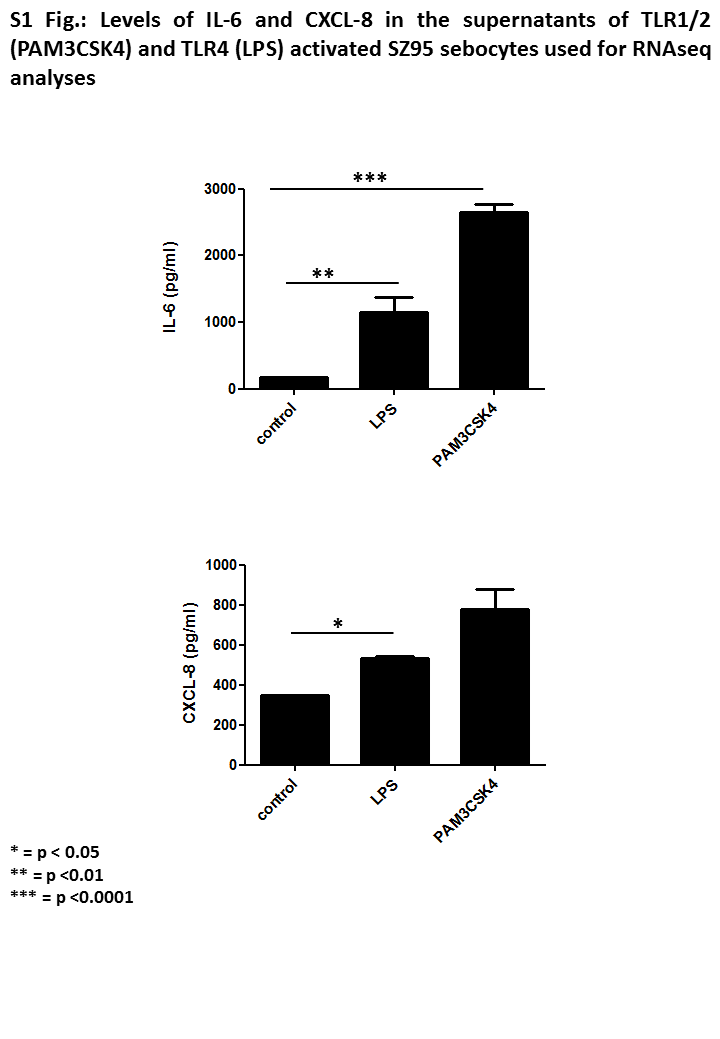

Supplement: S1 Fig — Protein levels of IL-6 and CXCL-8 were measured by ELISA as described in Materials and Methods. One-way ANOVA and Dunnett post-hoc test were used in the data analyses (n = 3); * = p < 0.05, ** = p <0.01, *** = p <0.0001. (TIF) [file pone.0198323.s001.TIF]

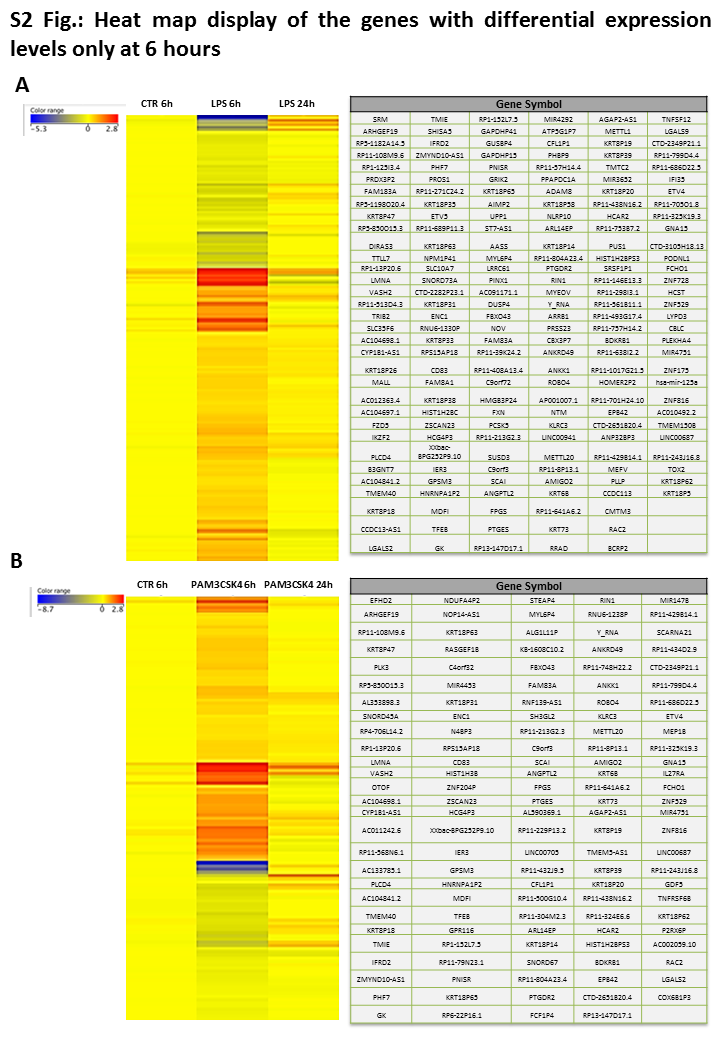

Supplement: S2 Fig — (TIF) [file pone.0198323.s002.TIF]

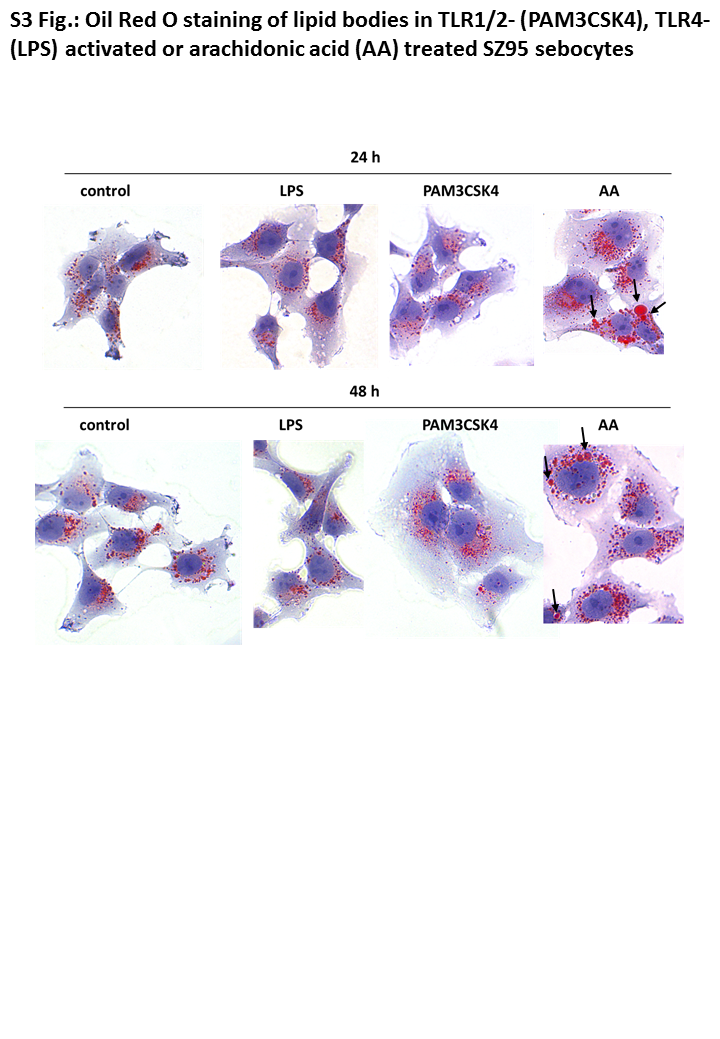

Supplement: S3 Fig — Oil Red O staining revealed no changes in the lipid body formation of TLR1/2- and TLR4-activated sebocytes neither at 24-hour nor at 48-hour time points. Arachidonic acid (AA) treatment inducing lipid body formation both in numbers and size was used as a positive control. At least three independent samples were stained per each treatment. (TIF) [file pone.0198323.s003.TIF]
