# Supplementary material for: Genome wide analysis of TLR1/2- and TLR4-activated SZ95 sebocytes reveals a complex immune-competence and identifies serum amyloid A as a marker for activated sebaceous glands
Source: PLoS One. 2018 Jun 21;13(6):e0198323. doi: 10.1371/journal.pone.0198323 (PMC6013244; doi:10.1371/journal.pone.0198323)
Supplement: S1 Table — Note the significant up-regulation in the expression levels of the cluster forming genes with the exception of ABCG1. (DOCX) [file pone.0198323.s004.docx]

**S1 Table: Genes involved in lipid metabolism with altered expression levels at 24 hours**

| **Gene ID** | **p (Corr)** | **p** | **Regulation ([LPS-24h] vs [control-24h])** | **Regulation ([PAM3CSK4-24h] vs [control-24h])** | **FC ([LPS-24h] vs [control-24h])** | **FC ([PAM3CSK4-24h] vs [control-24h])** | **Gene Symbol** | **Description** |
| --- | --- | --- | --- | --- | --- | --- | --- | --- |
| ENSG00000112972 | 3,00E-07 | 2,47E-10 | up | up | 1,940501 | 1,8111215 | HMGCS1 | 3-hydroxy-3-methylglutaryl-CoA synthase 1 (soluble) [Source:HGNC Symbol;Acc:5007] |
| ENSG00000167508 | 0,00400828 | 5,51E-05 | up | up | 1,8367671 | 1,8759025 | MVD | mevalonate (diphospho) decarboxylase [Source:HGNC Symbol;Acc:7529] |
| ENSG00000172893 | 8,04E-05 | 3,92E-07 | up | up | 1,7843913 | 1,6973399 | DHCR7 | 7-dehydrocholesterol reductase [Source:HGNC Symbol;Acc:2860] |
| ENSG00000258872 | 0,03842742 | 0,00133654 | up | up | 1,7506112 | 1,5828055 | FDPSP3 | farnesyl diphosphate synthase pseudogene 3 [Source:HGNC Symbol;Acc:3634] |
| ENSG00000001630 | 1,06E-05 | 3,17E-08 | up | up | 1,7238411 | 1,6097151 | CYP51A1 | cytochrome P450, family 51, subfamily A, polypeptide 1 [Source:HGNC Symbol;Acc:2649] |
| ENSG00000160285 | 2,02E-05 | 6,88E-08 | up | up | 1,7084069 | 1,6758851 | LSS | lanosterol synthase (2,3-oxidosqualene-lanosterol cyclase) [Source:HGNC Symbol;Acc:6708] |
| ENSG00000147383 | 2,17E-04 | 1,29E-06 | up | up | 1,6561968 | 1,5390469 | NSDHL | NAD(P) dependent steroid dehydrogenase-like [Source:HGNC Symbol;Acc:13398] |
| ENSG00000104549 | 1,55E-06 | 2,38E-09 | up | up | 1,6409988 | 1,5578208 | SQLE | squalene epoxidase [Source:HGNC Symbol;Acc:11279] |
| ENSG00000120437 | 3,06E-04 | 1,98E-06 | up | up | 1,6341988 | 1,5134673 | ACAT2 | acetyl-CoA acetyltransferase 2 [Source:HGNC Symbol;Acc:94] |
| ENSG00000130164 | 0,01610895 | 3,65E-04 | up | up | 1,6135386 | 1,5868397 | LDLR | low density lipoprotein receptor [Source:HGNC Symbol;Acc:6547] |
| ENSG00000147155 | 0,02271452 | 6,04E-04 | up | up | 1,6062952 | 1,6454473 | EBP | emopamil binding protein (sterol isomerase) [Source:HGNC Symbol;Acc:3133] |
| ENSG00000079459 | 0,00255268 | 2,85E-05 | up | up | 1,5306269 | 1,5032138 | FDFT1 | farnesyl-diphosphate farnesyltransferase 1 [Source:HGNC Symbol;Acc:3629] |
| ENSG00000186480 | 0,00477499 | 7,03E-05 | up | up | 1,5272634 | 1,5469906 | INSIG1 | insulin induced gene 1 [Source:HGNC Symbol;Acc:6083] |
| ENSG00000113161 | 9,64E-05 | 5,04E-07 | up | up | 1,5270901 | 1,3950703 | HMGCR | 3-hydroxy-3-methylglutaryl-CoA reductase [Source:HGNC Symbol;Acc:5006] |
| ENSG00000110921 | 0,0127067 | 2,57E-04 | up | up | 1,5245289 | 1,507515 | MVK | mevalonate kinase [Source:HGNC Symbol;Acc:7530] |
| ENSG00000169174 | 7,03E-06 | 1,99E-08 | up | up | 1,5035696 | 1,5236946 | PCSK9 | proprotein convertase subtilisin/kexin type 9 [Source:HGNC Symbol;Acc:20001] |
| ENSG00000116133 | 0,03379963 | 0,00110339 | up | up | 1,483221 | 1,4100499 | DHCR24 | 24-dehydrocholesterol reductase [Source:HGNC Symbol;Acc:2859] |
| ENSG00000078098 | 5,09E-04 | 3,69E-06 | up | up | 1,4752578 | 1,7346873 | FAP | fibroblast activation protein, alpha [Source:HGNC Symbol;Acc:3590] |
| ENSG00000067064 | 0,01114342 | 2,12E-04 | up | up | 1,3908613 | 1,3287097 | IDI1 | isopentenyl-diphosphate delta isomerase 1 [Source:HGNC Symbol;Acc:5387] |
| ENSG00000128284 | 1,42E-06 | 2,10E-09 | up | up | 1,3479447 | 1,5181383 | APOL3 | apolipoprotein L, 3 [Source:HGNC Symbol;Acc:14868] |
| ENSG00000100342 | 0,0017809 | 1,75E-05 | up | up | 1,2564268 | 1,4248303 | APOL1 | apolipoprotein L, 1 [Source:HGNC Symbol;Acc:618] |
| ENSG00000160179 | 0,00399403 | 5,47E-05 | down | down | -1,5349739 | -1,3811529 | ABCG1 | ATP-binding cassette, sub-family G (WHITE), member 1 [Source:HGNC Symbol;Acc:73] |
